# Genetically predicted serum testosterone and risk of gynecological disorders: a Mendelian randomization study

**DOI:** 10.3389/fendo.2023.1161356

**Published:** 2023-11-21

**Authors:** Benzheng Zhao, Zhenpeng Wang, Dongzhen Liu, Songling Zhang

**Affiliations:** Department of Obstetrics and Gynecology, The First Hospital of Jilin University, Changchun, China

**Keywords:** serum testosterone, sex hormone-binding globulin, gynecological disorders, Mendelian randomization, causal inference

## Abstract

**Background:**

Testosterone plays a key role in women, but the associations of serum testosterone level with gynecological disorders risk are inconclusive in observational studies.

**Methods:**

We leveraged public genome-wide association studies to analyze the effects of four testosterone related exposure factors on nine gynecological diseases. Causal estimates were calculated by inverse variance–weighted (IVW), MR–Egger and weighted median methods. The heterogeneity test was performed on the obtained data through Cochrane’s Q value, and the horizontal pleiotropy test was performed on the data through MR–Egger intercept and MR-PRESSO methods. “mRnd” online analysis tool was used to evaluate the statistical power of MR estimates.

**Results:**

The results showed that total testosterone and bioavailable testosterone were protective factors for ovarian cancer (odds ratio (OR) = 0.885, P = 0.012; OR = 0.871, P = 0.005) and endometriosis (OR = 0.805, P = 0.020; OR = 0.842, P = 0.028) but were risk factors for endometrial cancer (OR = 1.549, P < 0.001; OR = 1.499, P < 0.001) and polycystic ovary syndrome (PCOS) (OR = 1.606, P = 0.019; OR = 1.637, P = 0.017). dehydroepiandrosterone sulfate (DHEAS) is a protective factor against endometriosis (OR = 0.840, P = 0.016) and premature ovarian failure (POF) (OR = 0.461, P = 0.046) and a risk factor for endometrial cancer (OR= 1.788, P < 0.001) and PCOS (OR= 1.970, P = 0.014). sex hormone-binding globulin (SHBG) is a protective factor against endometrial cancer (OR = 0.823, P < 0.001) and PCOS (OR = 0.715, P = 0.031).

**Conclusion:**

Our analysis suggested causal associations between serum testosterone level and ovarian cancer, endometrial cancer, endometriosis, PCOS, POF.

## Introduction

Testosterone is an essential sex hormone in women. It can be directly synthesized by the ovary or transformed by androstenedione and dehydroepiandrosterone (DHEA), known as preandrogens, which are produced by the ovary and adrenal glands (1). At the age of 6-8 years, the testosterone level in women begins to rise due to the maturation of the zona reticularis. In the reproductive period, it further increases due to the beginning of ovulation, peaks at the age of 20-25 years, and then decreases gradually (1, 2). Circulating testosterone is mainly bound to plasma proteins, most of which are bound to sex hormone-binding globulin (SHBG) and a small part to albumin. In peripheral target tissues, testosterone is converted by 5α-reductases into dihydrotestosterone (DHT), which exhibits stronger binding to androgen receptors and higher biological activity (1).

Although the level of testosterone in women is lower than that in men, this hormone plays an important role in maintaining normal functions related to women’s psychology and physiology, among others. Several studies have indicated that female testosterone levels are related to type 2 diabetes (3), psychiatric disorders (4), sexual function (1), cardiovascular disease (5) and bone health (6). At present, most studies on the influence of testosterone on gynecological diseases focus on androgen receptor (AR) and androgen metabolic pathway. AR is a member of the nuclear receptor superfamily and participates in cell proliferation, differentiation, apoptosis, metabolism, secretion and other regulatory functions. When androgen combines with AR, it can start gene transcription through classical or non-classical pathways. In the classical pathway, AR can combine with androgen response elements (AREs) to start gene transcription. In the non-classical pathway, AR can activate multiple signal pathways such as MAPK/ERK (Mitogen activated protein kinase/extracellular regulated protein kinase), mTOR (Mammalian target of rapamycin), Src (Tyrosine protein kinase), c-Myc (bHLH transfer factor). These pathways further target EGFR (Epidermal growth factor receiver), IGF-I (Insulin growth factor I), TGF-β (transforming growth factor-β) and other genes to perform biological functions (7). A variety of enzymes including SRD5A1 (steroid 5 alpha reductase 1), StAR (steroidal acid regulatory protein), CYP11A1 (cyclochrome P450 family 11 subfamily A member 1), CYP17A1 (cyclochrome P450 family 17 subfamily A member 1) and AKR1C3 (aldo-keto reduce family 1 member C3) participate in the process of androgen metabolism and transformation and further play biological functions in a variety of ovarian and endometrial pathological processes (8, 9). However, the epidemiological evidence of testosterone on gynecological diseases is still insufficient or controversial. For example, a pooled analysis of 7 nested case control studies on ovarian cancer showed that the level of testosterone was positively correlated with the risk of epithelial ovarian cancer (EOC) and that high concentrations of testosterone and androstenedione were associated with an increased risk of endometrioid and mucinous tumors (10). In contrast, another European prospective study showed that there was no correlation between serum androgens or SHBG concentration and ovarian cancer risk (11). Few reports have explored the correlation between testosterone and the risk of cervical cancer. Studies on nonneoplastic diseases, such as polycystic ovary syndrome (PCOS), focus on the symptoms of hyperandrogenemia, but whether a high concentration of testosterone is the cause of PCOS remains controversial (12). Therefore, new insights are needed to clarify the causal relationship between testosterone and a variety of malignant or nonmalignant female reproductive system diseases.

Mendelian randomization (MR) is a statistical method for causal inference using genetic variants as instrumental variables (IV). Since genetic variation results from random splits and combinations in the process of gamete formation and is not related to the acquired environmental confounders, the difference in a certain outcome between the population with and the one without the variation can be attributed to the variation in exposure factors to eliminate the interference of confounding factors (13). Because the development of genetic variation precedes the occurrence and change in the level of environmental exposure, confounding, and disease outcome, the exposure variation explained by genetic variation as an IV of exposure also precedes the outcome, and the reverse causality problem is also excluded (14). Therefore, MR research has clear advantages over traditional observational studies and is more convenient and cost-efficient than traditional randomized controlled trials (RCTs). In recent years, with the rapid development of gene sequencing technology, many genetic variants closely related to specific traits have been found. With the publication of many large sample genome-wide association studies (GWASs), the research effectiveness of MR has also been greatly improved. In this study, we used MR to analyze the causal relationship between exposure factors, such as total testosterone (TT), bioavailable testosterone (BioT), dehydroepiandrosterone sulfate (DHEAS) and SHBG, and female reproductive system diseases, including ovarian cancer, endometrial cancer, cervical cancer, endometriosis, polycystic ovary syndrome (PCOS), premature ovarian failure (POF), cervical polyps and uterine fibroids.

## Materials and methods

This study used summary level data from the published studies and publicly available GWASs, with ethical approvals obtained from their respective institutional review boards and informed consent provided by their participants.

### Summary statistics of four androgen-related risk factors from genome-wide association studies

Through the MRCIEU GWAS database (https://gwas.mrcieu.ac.uk/), we searched for the TT, BioT, DHEAS and SHBG of four androgen-related risk factors and summarized their GWAS data. The GWAS data on TT, BioT and SHBG are all from the same UK Biobank (UKB)-based study, which detected the TT level of 199569 European women, the BioT level of 180386 European women and the SHBG level of 214989 European women and involved a total of 12321875 SNP sites. GWAS data for DHEAS were obtained from a metabolism-related GWAS of 7793 European adults involving 2545593 SNP sites (15) (**Table 1**).

**Table 1:** Summary of exposures factors.

Exposure	NSNP	Unit	Sample	R^2^(%)	F	GWAS ID
TT	114/135	SD	199569	3.97/4.63	72.37/71.66	ieu-b-4864
Bio-T	125/150	SD	180386	4.94/5.67	82.85/79.94	ieu-b-4869
DHEAS	10	SD	9722	6.49	1384.51	ebi-a-GCST004941
SHBG	197	SD	214989	8.47	93.63	ieu-b-4870

NSNP, number of SNPs; F, F statistics; GWAS ID, GWAS data ID of MRCIEU GWAS database; R^2^, phenotype variance explained by genetics.

### Summary statistics of genome-wide association studies related to nine female reproductive system diseases and their histological subtypes

Similarly, we summarized and analyzed GWASs of 9 female reproductive system diseases and their histological subtypes through the MRCIEU GWAS database. GWAS data on ovarian cancer and its four histological subtypes (serous, mucinous, endometrioid and clear cell carcinoma) were obtained from the international Ovarian Cancer Association Consortium (OCAC) (16). The total sample sizes (cases, controls) of serous ovarian cancer, mucinous ovarian cancer, endometrioid ovarian cancer and clear cell ovarian cancer were 54,990 (14049 cases, 40941 controls), 42358 (1417 cases, 40941 controls), 43751 (2810 cases, 40941 controls) and 42307 (1,366 cases, 40,941 controls), respectively.

GWAS data on endometrial cancer and its two histological subtypes (endometrioid histology and non-endometrioid histology) are from a meta GWAS (17), which included 17 GWAS studies from the Endometrial Cancer Association Consortium (ECAC), the Epidemiology of Endometrial Cancer Consortium (E2C2) and the UKB; all subjects were of European descent. The total number of endometrial cancer samples was 121885 (12906 cases, 108979 controls), including 54884 (8758 cases, 46126 controls) of endometrioid histology and 36677 (1230 cases, 35447 controls) of non-endometrioid histology, and involved 8974630 SNP sites.

GWAS data on cervical cancer and cervical polyps are from the Medical Research Council Integrated Epidemiology Unit (MRC-IEU) consortium based on UKB self-reported cervical cancer and polyp of cervix uteri data. A total of 463010 European women were included in the cervical cancer study, including 462933 European women (1889 patients with cervical cancer and 461044 controls). A total of 463010 European women were included in the cervical polyp study, including 2307 patients with cervical polyps and 460703 controls. A total of 9851867 SNP sites were analyzed in both studies.

GWAS data on ovarian cysts, endometriosis, PCOS and POF were all from Finngen (https://www.finngen.fi/en), and all subjects were of European descent. The ovarian cyst study included 10848 cases and 68969 controls, involving 16377648 SNP sites; the endometriosis study included 8288 cases and 68969 controls, involving 16377306 SNP sites; the PCOS study included 642 cases and 118228 controls, involving 1637966 SNP sites; and the POF study included 254 cases and 118228 controls, involving 1637967 SNP sites.

GWAS data on uterine fibroids came from a study based on UKB self-reported uterine fibroid data by the Neale Lab Consortium. A total of 337159 European women were included in the study (5168 patients with uterine fibroids and 331991 controls). A total of 10894596 SNP sites were analyzed (**Table 2**).

**Table 2:** Summary of outcomes.

Outcome	Ncontrol	Ncase	GWAS ID
**Ovarian cancer**	40941	25,509	ieu-a-1120
Serous	40941	14,049	ieu-a-1228
Mucinous	40941	1,417	ieu-a-1123
Endometrioid	40941	2,810	ieu-a-1125
Clear cell	40941	1,366	ieu-a-1124
**Endometrial cancer**	108,979	12,906	ebi-a-GCST006464
Endometrioid	46,126	8,758	ebi-a-GCST006465
Non-endometrioid	35,447	1,230	ebi-a-GCST006466
**Cervical cancer**	461,044	1,889	ukb-b-8777
**Ovarian cyst**	68,969	10,848	finn-b-N14_OVARYCYST
**Endometriosis**	68,969	8,288	finn-b-N14_ENDOMETRIOSIS
**POF**	118,228	254	finn-b-E4_OVARFAIL
**PCOS**	118,228	642	finn-b-E4_POCS
**Cervical polyp**	460,703	2,307	ukb-b-19110
**Uterine fibroids**	331,991	5,168	ukb-a-91

### MR design and statistical analysis

**Figure 1:**
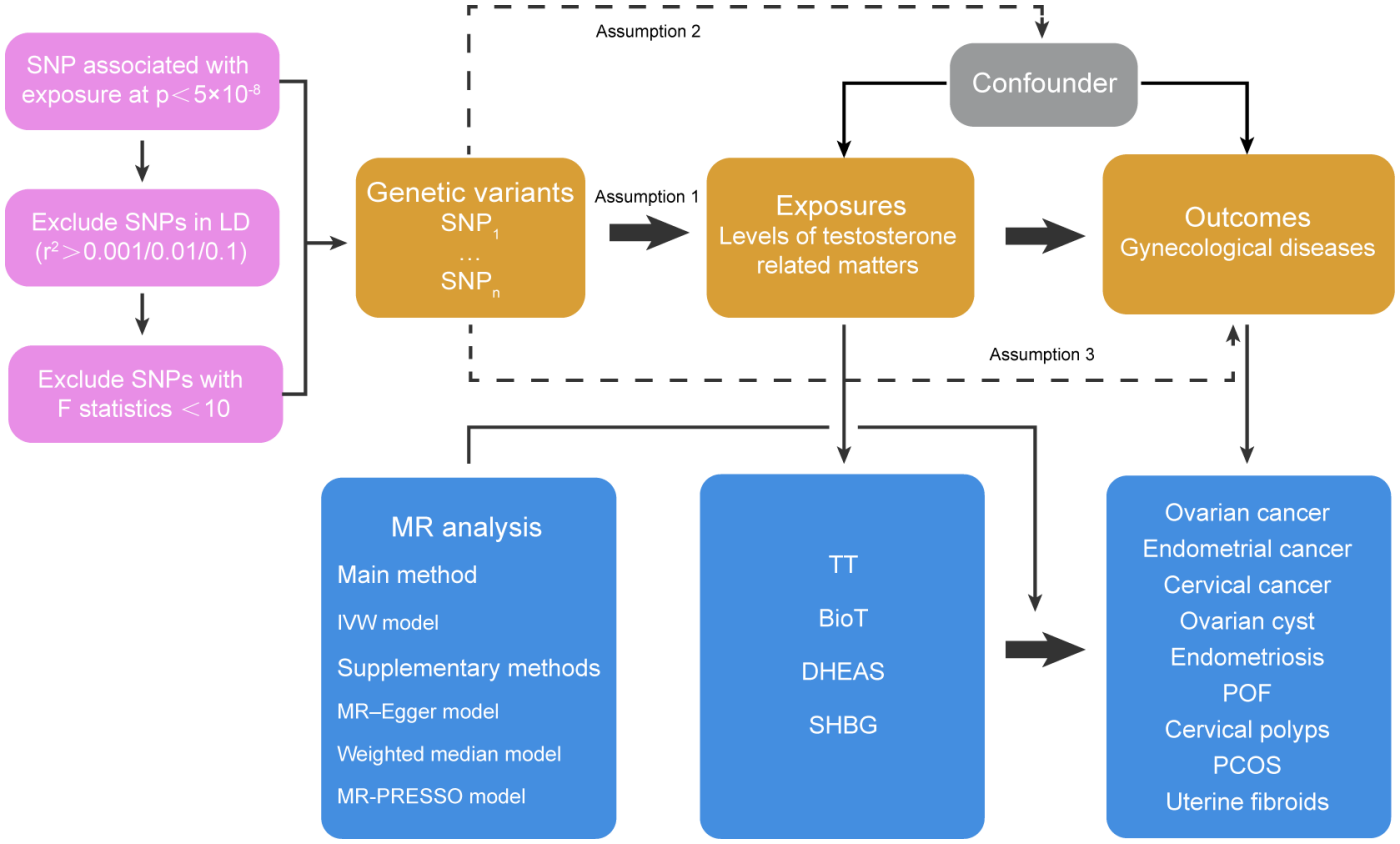
Basic assumptions of Mendelian randomization and main design of this study. Assumption 1 indicates that genetic variants as instrumental variables should be closely related to risk factors; Assumption 2 indicates that the instrumental variables should not be related to potential confounding factors, and Assumption 3 indicates that the instrumental variables should only affect the risk of outcomes through risk factors rather than other pathways. SNP, single nucleotide polymorphisms; LD, linkage disequilibrium; IVW, inverse-variance weighted; TT, total testosterone; BioT, bioavailable testosterone; DHEAS, dehydroepiandrosterone sulfate; SHBG, sex hormone-binding globulin; POF, premature ovarian failure; PCOS, polycystic ovary syndrome.

GWAS P < 5 × 10^-8^ and minor allele frequency > 0.01 were used as criteria to screen SNPs that can represent four risk factors: TT, BioT, DHEAS and SHBG. To remove the SNPs with linkage disequilibrium (LD), we screened the specific SNPs TT, BioT and SHBG with R^2^ < 0.001 and kb = 10000 as the thresholds. Because the number of DHEAS SNPs screened was too small to complete the subsequent analysis if using R^2^ < 0.001 and kb = 10000 as the criteria, we relaxed the screening criteria to R^2^ < 0.1 and kb = 5000. The weak instrument bias of each group of SNPs was evaluated by calculating F statistics (F = beta^2^/se^2^), and SNPs with low statistical power were excluded (F statistics < 10) (18). Finally, the degree of interpretation of each exposure factor was evaluated by calculating the F statistics of all specific SNPs in each group. In the process of MR analysis, we analyzed the effects of four androgen-related exposure factors on nine female reproductive system diseases and their subtypes by inverse variance–weighted (IVW), MR–Egger and weighted median methods through the R package “TwoSampleMR”. The heterogeneity test was performed on the obtained data through Cochrane’s Q value, and the horizontal pleiotropy test was performed on the data through MR–Egger intercept (19) and MR-PRESSO methods (20). Data exhibiting heterogeneity were reanalyzed with a random effect model, and data exhibiting horizontal pleiotropy had the outlying SNP eliminated and were reanalyzed through the R package “MR-PRESSO” (**Figure 1**). To deal with potential conflicts of MR assessments, we used multivariable Mendelian randomization (MVMR) method to analyze body mass index (BMI, GWAS ID: ieu-a-2) and outcome data with significant differences (p<0.05) in our results. We established a multiple testing significance threshold defined as p < 0.05/n (n = 4, the effective number of exposures). Finally, we used the mRnd online analysis tool (https://cnsge.nomics.shiny.apps.IO/MRND/) to evaluate the statistical power of MR estimates. All statistical analyses and data visualization were performed using R software 4.1.0.

## Results

**Figure 2:**
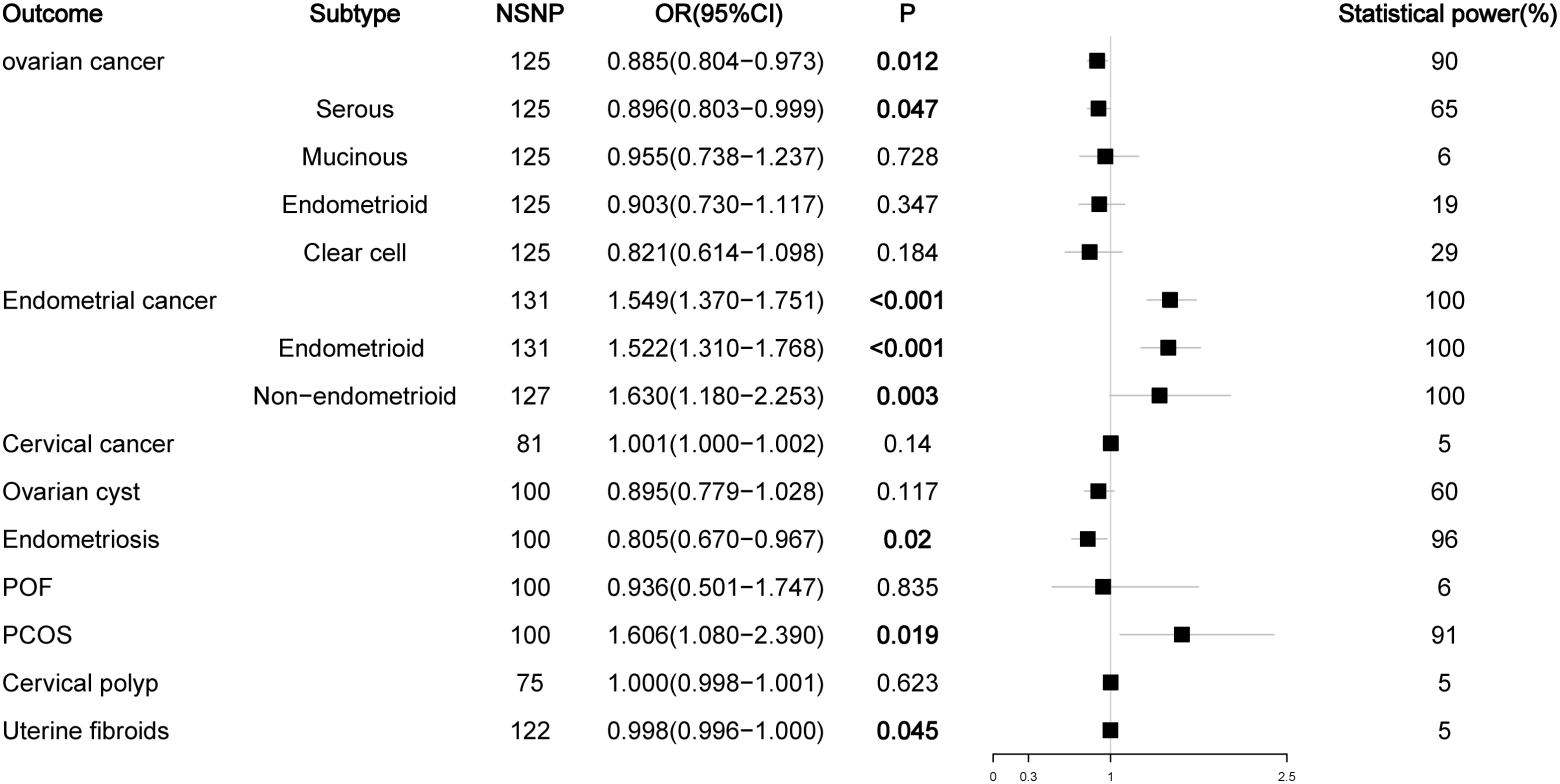
Associations of total testosterone with gynecological diseases (IVW method). NSNP, number of single nucleotide polymorphisms; OR, odds ratio; 95%CI, 95% confidence interval; P, p value.

**Figure 3:**
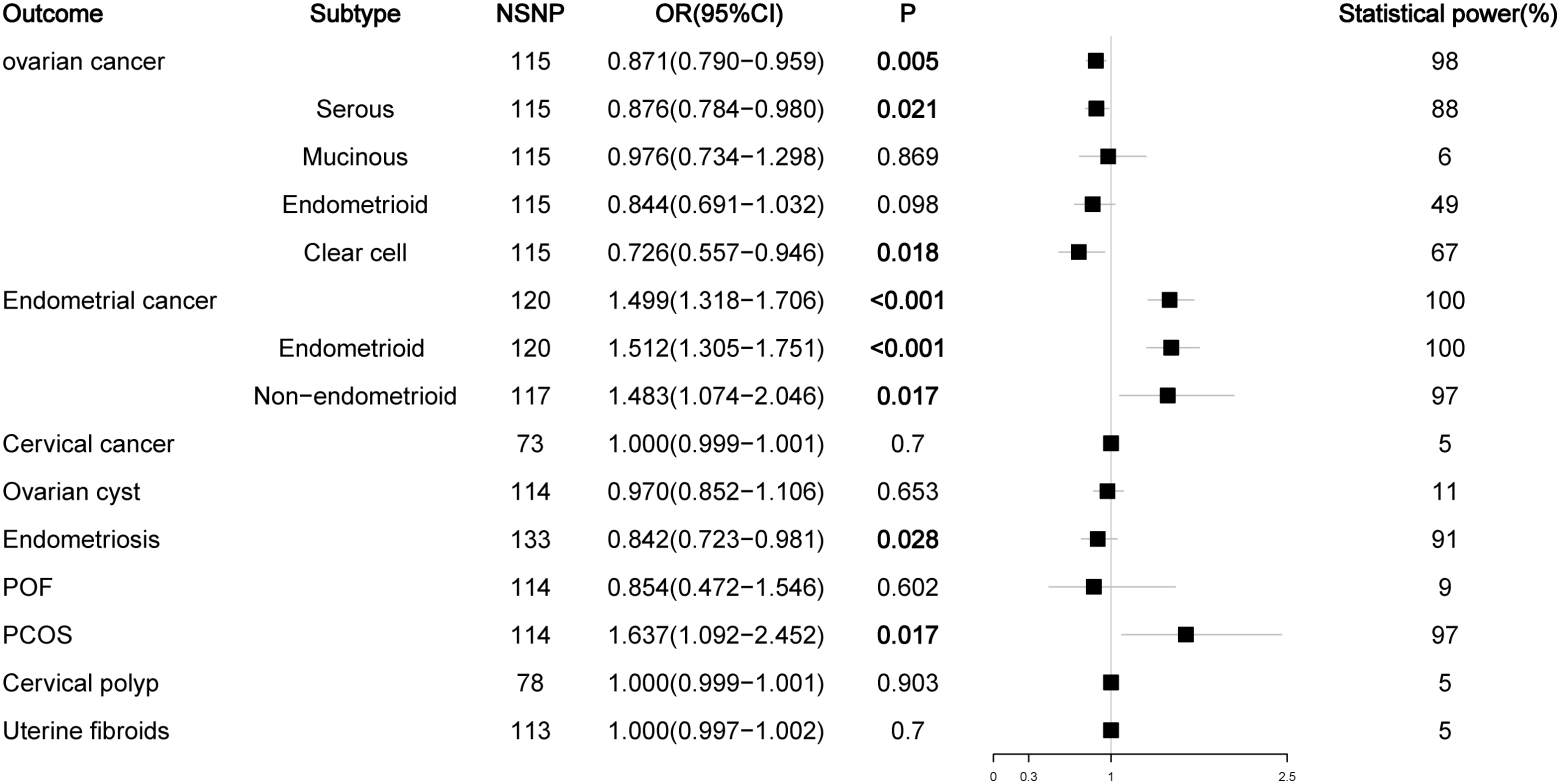
Associations of bioavailable testosterone with gynecological diseases (IVW method).

**Figure 4:**
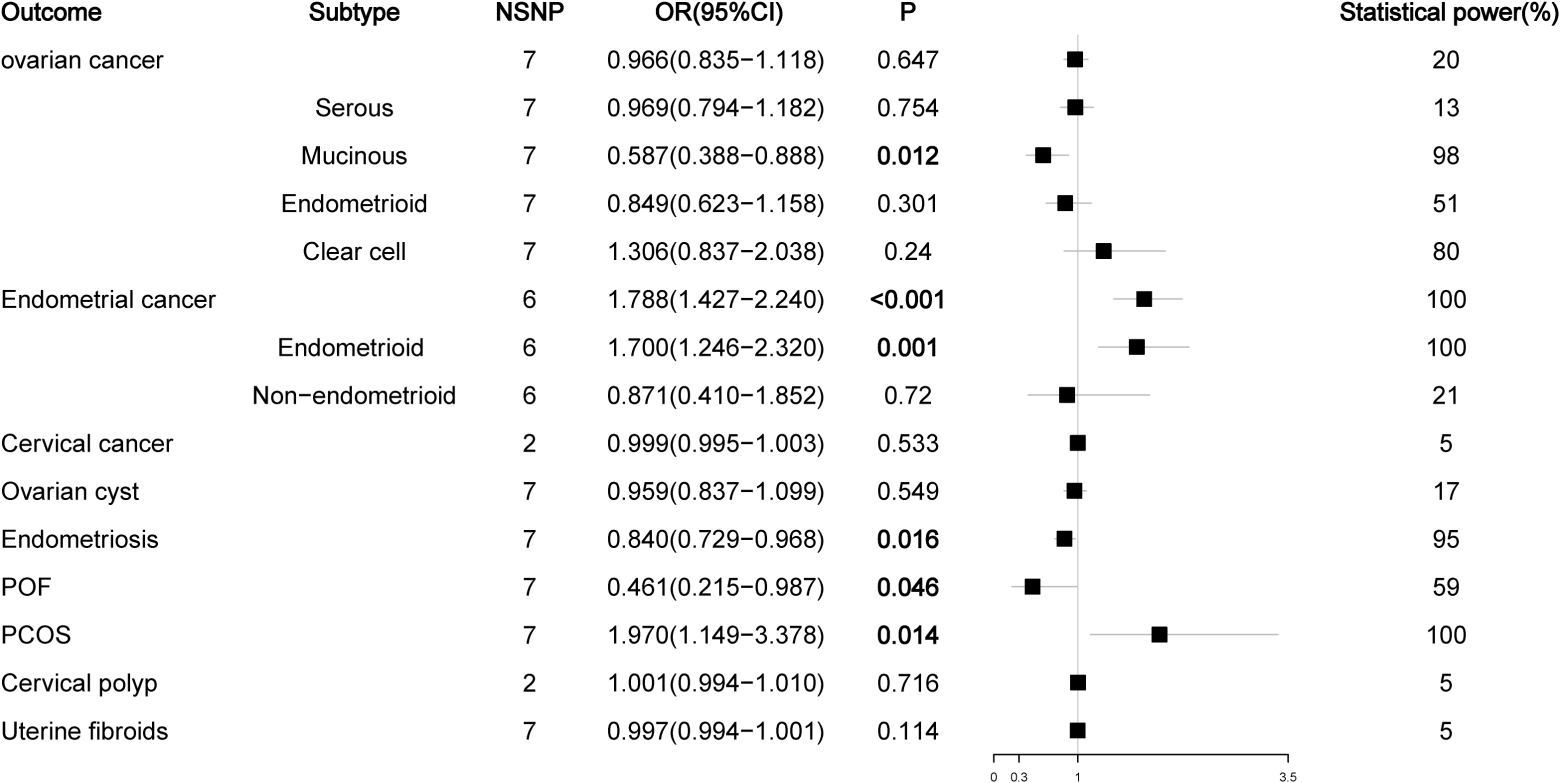
Associations of dehydroepiandrosterone sulfate with gynecological diseases (IVW method).

**Figure 5:**
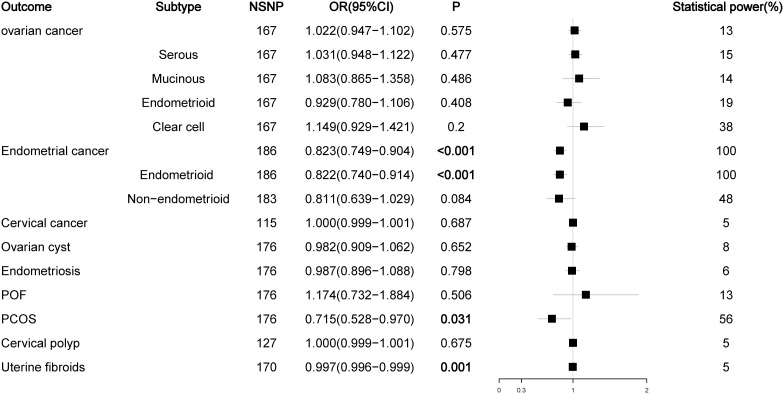
Associations of sex hormone-binding globulin with gynecological diseases (IVW method).

The number of SNPs ranged from 2 to 186, and the explained variables varied from 3.97% to 8.47%. The F statistics of each SNP and the general F statistics were greater than the empirical threshold 10. The MR analysis results of all groups have passed the heterogeneity test and horizontal pleiotropy test (**Tables S1-S4**), and the statistical power of all outcomes was shown in **Figures 2**–**5**, The BMI corrected p-values for significant differences in results are shown in **Table S5**.

### Effect of total testosterone on gynecological diseases

We first analyzed the relationship between TT levels and the risk of nine female reproductive system diseases and their histological subtypes. The results showed that high levels of TT decreased the risk of ovarian cancer (OR = 0.892, P = 0.025) and endometriosis (OR = 0.805, P = 0.020) but increased the risk of endometrial cancer (OR = 1.505, P < 0.001) (mainly the endometrioid histological subtype (OR = 1.490, P < 0.001)) and PCOS (OR = 1.606, P = 0.019). Our initial results did not find a relationship between TT levels and the risk of four histological subtypes of ovarian cancer and non-endometrioid histological subtypes of endometrial cancer (P > 0.05), but the P values in the analyses of TT levels and serous ovarian cancer (OR = 0.890, P = 0.052) or non-endometrioid endometrial cancer (OR = 1.416, P = 0.055) were close to the critical value. Considering that this may be due to the lack of SNPs as a result of our overly strict screening criteria for LD to obtain testosterone data, we relaxed the LD screening criteria to R^2^ < 0.01 and kb = 5000 and reanalyzed the impact of TT levels on the outcome of ovarian and endometrial cancer. The results showed that high levels of TT reduced the risk of ovarian cancer (OR = 0.885, P = 0.012) and serous ovarian cancer (OR = 0.896, P = 0.047) and increased the risk of endometrial cancer (OR = 1.549, P < 0.001) and all its subtypes (endometrioid histology: OR = 1.522, P < 0.001; non-endometrioid histology: OR = 1.630, P = 0.003). In addition, we noted that high TT levels slightly reduced the risk of uterine fibroids (OR = 0.998, P = 0.045). Because the OR value was too close to the critical value (OR = 1.000), we did not suggest that TT could have an impact on the risk of uterine fibroids (**Figure 2**; **Figure S1**).

### Effect of bioavailable testosterone on gynecological diseases

Since most circulating testosterone is tightly bound to SHBG and a small part is bound to albumin, only unbound or free testosterone has biological activity in target tissues (21, 22). Therefore, we analyzed the effect of BioT levels on malignant or nonmalignant gynecological diseases. The results showed that high levels of BioT could also reduce the risk of ovarian cancer (OR = 0.871, P = 0.005) and increase the risk of endometrial cancer (OR = 1.499, P < 0.001) (endometrial subtype: OR = 1.512, P < 0.001; non-endometrial subtype: OR = 1.483, P = 0.017) and PCOS (OR = 1.637, P = 0.017). In contrast to the previous results of TT, BioT not only reduced the risk of serous ovarian cancer (OR = 0.876, P = 0.021) but also reduced the risk of clear cell ovarian cancer (OR = 0.726, P = 0.018). In addition, after readjusting the LD screening criteria (R^2^ < 0.01, kb = 5000), high levels of BioT could also reduce the risk of endometriosis (OR = 0.842, P = 0.028) (**Figure 3**; **Figure S2**).

### Effect of DHEAS and SHBG on gynecological diseases

As a prohormone predictor, DHEA or its sulfate form DHEAS will naturally affect the level of testosterone. Because SHBG can bind to testosterone, SHBG levels are negatively correlated with BioT levels, so SHBG can be used as a marker to evaluate androgen activity. Therefore, we analyzed the effects of DHEAS and SHBG levels on gynecological diseases. In the analysis of DHEAS, we found that there was no causal relationship between DHEAS level and ovarian cancer or its serous subtype (P > 0.05), but high levels of DHEAS could reduce the risk of mucinous ovarian cancer (OR = 0.587, P < 0.012). For endometrial cancer, high levels of DHEAS remained a risk factor for endometrial cancer (OR= 1.788, P < 0.001) and its endometrioid histological subtype (OR = 1.700, P = 0.001), but there was no causal relationship between DHEAS and non-endometrioid endometrial cancer (OR = 0.871, P = 0.720). For nonmalignant gynecological diseases, high DHEAS levels reduced the risk of endometriosis (OR = 0.840, P = 0.016) and increased the risk of PCOS (OR= 1.970, P = 0.014). Interestingly, high levels of DHEAS could reduce the risk of POF (OR = 0.461, P = 0.046); this result was not observed in the previous TT and BioT findings (**Figure 4**; **Figure S3**). The results of the SHBG analysis showed that SHBG also no longer had a causal relationship with the risk of ovarian cancer and endometriosis (P > 0.05), while high-level SHBG could reduce the risk of endometrial cancer (OR = 0.823, P < 0.001) and its endometrioid histological subtype (OR = 0.822, P < 0.001) but no longer had a causal relationship with non-endometrioid endometrial cancer (OR = 0.811, P = 0.084). In addition, high levels of SHBG could still reduce the risk of PCOS (OR = 0.715, P = 0.031) (**Figure 5**; **Figure S4**).

## Discussion

EOC is the deadliest gynecological cancer. Every year, 230000 women around the world are diagnosed with ovarian cancer and 150000 women die of the disease (23). Many studies have extensively discussed the etiology of ovarian cancer. For example, many studies believe that the continuous destruction and repair of ovarian epithelial cells caused by ovulation may be the cause of ovarian cancer. The inhibition of ovulation by the use of oral contraceptives or by pregnancy, for example, reduces the risk of ovarian cancer. Other factors, such as a family history of EOC, smoking, benign gynecological diseases (including endometriosis, polycystic ovary syndrome and pelvic inflammation), and even the use of talcum powder, are considered to be risk factors for epithelial ovarian cancer (24). MR studies on ovarian cancer have also been reported in many studies. For example, Gao et al. and Dixon et al. considered that a higher genetically-predicted BMI was associated with an increased risk of ovarian cancer (25, 26). Ong et al. pointed out that high levels of vitamin D could increase the risk of ovarian cancer (27). Haycock et al. suggested that increased telomere length was associated with a higher risk of ovarian cancer (28). Yang et al. pointed out that an increase in age at menarche would reduce the risk of ovarian cancer (29). In our MR study, we found that high levels of TT and BioT were protective factors against ovarian cancer. Previously, we mentioned that the existing conclusions of RCTs or case–control studies on the correlation between testosterone and ovarian cancer risk were still controversial. We thought this might be because previous studies had fewer cases, and the interference of environmental exposure and confounding factors could not be excluded in traditional epidemiological studies. In addition, ovarian cancer is a heterogeneous disease; its different histological subtypes show different molecular characteristics and at the same time have different risk factors (30). Considering this, we also analyzed four histological subtypes of ovarian cancer. The results showed that TT and BioT were also protective factors against serous ovarian cancer. Because serous ovarian cancer is the most common subtype of ovarian cancer, this result was also in line with our expectation. Surprisingly, the BioT analysis results showed that high levels of BioT could also reduce the risk of clear cell ovarian cancer, while high levels of DHEAS could reduce the risk of mucinous ovarian cancer. The mechanism involved still needs to be further studied.

Endometrial cancer is the most common gynecologic cancer in the world, and its incidence continues to rise. Previous studies have shown that increasing age, high concentrations of estrogens postmenopause, metabolic syndrome (obesity, diabetes), long-term use of tamoxifen, progestogens and a family history of endometrial cancer are risk factors for endometrial cancer (31). MR studies on endometrial cancer also pointed out that high levels of low-density lipoprotein (LDL) cholesterol were associated with a reduced risk of endometrial cancer (32), while high levels of cortisol were associated with an increased risk of endometrial cancer (33). In our MR study, we found that high levels of TT, BioT and DHEAS were associated with a high risk of endometrial cancer. High levels of TT and BioT could increase the risks of both endometrioid endometrial cancer and non-endometrioid endometrial cancer, while SHBG was considered to be a protective factor for endometrial cancer. Because SHBG was negatively correlated with testosterone levels, these conflicting results could accurately prove the effect of testosterone on the risk of endometrial cancer. Studies have suggested that hyperandrogenemia in premenopausal women might reduce their resistance to estrogen-induced endometrial proliferation by inducing chronic anovulation and progesterone deficiency, thereby increasing the risk of endometrial cancer. Postmenopausal women might have high androgen levels due to obesity and other reasons; androgens in adipose tissue are transformed into estrogen by aromatase to promote abnormal endometrial proliferation and finally increase the risk of endometrial cancer. This might be the reason why high levels of testosterone could increase the risk of endometrial cancer.

Endometriosis refers to the presence of endometrioid tissue outside the uterine cavity. Patients with endometriosis usually have symptoms such as chronic pelvic pain, dysmenorrhea, difficult sexual intercourse and infertility, which seriously affect the psychological and physical health of women. At the same time, the fact that it recurs often also causes a huge economic burden to society. Studies have shown that low birth weight, an early age at menarche, a short menstrual cycle, low body mass index, low waist-to-hip ratio and low parity are risk factors for endometriosis (34). An MR analysis also showed that reduced weight and BMI could increase the risk of endometriosis (35). Our MR analysis showed that high levels of TT, BioT and DHEAS could reduce the risk of endometriosis. Although some studies have shown that there is abnormal expression of testosterone metabolism related genes CYP11A1, CYP17A1, HSD3B2 in endometriosis patients or endometriosis lesions (9), there is no direct epidemiological evidence to show the correlation between testosterone levels and the occurrence of endometriosis. This study will provide a new research strategy for the etiology of endometriosis.

PCOS is a heterogeneous disease characterized by hyperandrogenism, ovarian dysfunction and/or polycystic ovarian morphology (PCOM). Although the etiology of PCOS is not clear, studies have shown that a family history of PCOS, obesity, premature pubarche, hyperinsulinemia and high androgen levels are all associated with a high risk of PCOS (36). Previous MR studies on PCOS also pointed out that smoking (37) and age-related obesity (38) were risk factors for PCOS. Our MR analysis results showed that high levels of TT, BioT and DHEAS could increase the risk of PCOS, while high levels of SHBG reduced the risk of PCOS. This result was similar to the previous results of endometrial cancer. The opposite effect of SHBG could also confirm the important impact of testosterone on the risk of PCOS. Regarding the relationship between testosterone and PCOS, most studies focus more on testosterone as the key clinical feature of PCOS than on the etiology. Therefore, our research will provide a theoretical basis to consider testosterone the etiological agent of PCOS, but the mechanism involved in this relationship needs to be further studied.

Patients with POF usually reach menopause before the age of 40 as a result of the premature cessation of ovarian function, and the diagnosis of POF is often based on an increase in FSH levels to the menopausal range (typically above 40 IU/L). The possible causes of POF include genetic abnormalities, autoimmune ovarian injury, iatrogenic ovarian injury (radiotherapeutic or chemotherapeutic) and environmental factors (virus infection, toxins, etc.) (39). At present, there is no MR study on POF. Our results showed that high levels of DHEAS could reduce the risk of POF. In a case-control study, Barad et al. found that DHEA could increase the level of active oocytes and reduce their rate of atresia (40). We think this may be the reason why DHEAS can reduce the morbidity of POF. Meanwhile, a case report from Mamas et al. noted that five POF patients had reduced FSH levels and successfully became pregnant after receiving DHEA treatment. This also confirmed that DHEA might be a protective factor against POF.

Our MR analysis results highly reveal the close relationship between androgen and ovarian cancer, endometrial cancer, endometriosis, PCOS. These results make us believe that ovarian cancer, endometrial cancer, endometriosis and PCOS are androgen related diseases. In addition, the causal relationship between DHEAS and POF also provides a reference for us to understand the etiology of POF. In order to find out the potential molecular mechanism of androgen on the above five gynecological diseases, we analyzed the SNP sites involved in our MR analysis. The results showed that SNP sites such as AKR1C2 (rs116900756), AKR1D1 (rs9642092), CYP11B1 (rs1134095), CYP3A7 (rs45446698) appeared simultaneously in the MR analysis results of TT and BioT on ovarian cancer, endometrial cancer, endometriosis, PCOS and POF. AKR1C2 and AKR1D1, similar to the AKR1C3 mentioned above, are members of the aldosterone reductase superfamily, CYP11B1 and CYP3A7, similar to CYP11A1 and CYP17A1, are members of the cytochrome P450 family. These genes are all related to the metabolic regulation of steroid hormones, and are involved in the occurrence and development of prostate cancer (41–43), liver cancer (42, 44, 45), breast cancer (46–48), non-alcoholic fatty liver (49) and other benign or malignant diseases. Therefore, we believe that these genes may be potential targets of androgen affecting the outcome of gynecological diseases, and further research is needed to clarify their relationship. In addition, we found that all SNP sites involved in our research results contained CMIP (rs12447774) gene, which suggests that the immune cell regulation induced by c-Maf protein may participate in the influence of androgen on the occurrence and development of ovarian cancer, endometrial cancer, endometriosis, PCOS and POF.

This study has several clear strengths. The GWAS data of exposure and outcome used by us are from published high-quality studies or authoritative institutions, and the sample sizes of these studies were also large, thus preventing the insufficient statistical power caused by limited sample size; therefore, it has high reliability. In addition, the study population of all GWAS data is of European descent, and the TT and BioT data are all from European women, thus preventing errors caused by comparing different population subgroups and sexes. In the process of our MR analysis, we also had strict criteria for the screening of IV SNPs. The calculation of F statistics to evaluate the degree of interpretation of exposure factors, the use of IVW, MR-Egger and weighted median methods to analyze the results of MR analysis, and the use of different models or methods to correct the results with heterogeneity or horizontal pleiotropy makes our results more objective and accurate. Finally, we also tested the statistical validity of all MR analyses, which makes our results highly reliable.

Our research also has several deficiencies. Although we used the MR–Egger intercept and MR-PRESSO methods to remove the horizontal pleiotropy of MR analysis results as much as possible, it still cannot be completely removed, which will bias our results. As mentioned above, the GWAS data of TT and BioT are all women of European descent, but the GWAS data of DHEAS and SHBG are not classified by sex. Because there are vast differences in circulating testosterone levels between men and women, this may have a great impact on our results. In addition, since all the populations involved in our MR analysis are of European origin, our results cannot be extrapolated to other populations. All our MR analyses were performed using the two-sample MR method, which is only suitable for causal inference where there is a linear relationship between exposure and outcome rather than a nonlinear relationship. Although the GWAS data we used had a large sample size, for some outcome variables, such as POF and PCOS, the number of cases included in the GWAS data was small, which may have led to “false-positive” or “false-negative” results. In addition, because there are few instrumental variables for preliminary screening of DHEAS data, considering the possible impact of the “winner’s curse” (50), we lowered the screening criteria of the DHEAS IV SNPs, which may have caused some SNPs with LD to affect the results and led to the emergence of “false-positive” results. Finally, we failed to find other high-quality GWAS data to repeatedly verify our results, which makes it impossible to verify the repeatability of our results. We also hope to have more relevant studies to verify the reliability of our results in the future.

In conclusion, Our MR analysis showed that TT and BioT are protective factors for ovarian cancer and endometriosis, while they were risk factors for endometrial cancer and PCOS. DHEAS was a protective factor for mucinous ovarian cancer and endometriosis and a risk factor for endometrial cancer and PCOS. In addition, DHEAS was also a protective factor for POF. High levels of SHBG could reduce the risk of endometrial cancer and PCOS. This study will provide evidence for testosterone as a target for prevention and treatment of ovarian cancer, endometrial cancer, endometriosis, PCOS and POF.

## Data availability statement

The original contributions presented in the study are included in the article/**Supplementary Material**. Further inquiries can be directed to the corresponding author.

## Ethics statement

This study used summary level data from the published studies and publicly available GWASs, with ethical approvals obtained from their respective institutional review boards and informed consent provided by their participants. The studies were conducted in accordance with the local legislation and institutional requirements. The participants provided their written informed consent to participate in this study.

## Author contributions

SZ: Conceptualization; BZ: Methodology, Software; BZ, ZW: Formal analysis, Investigation; BZ, DL: Resources, Writing - Original Draft; SZ, BZ, ZW, DL: Writing - Review and Editing. All authors contributed to the article and approved the submitted version.
