# Supplementary material for: Genetically predicted serum testosterone and risk of gynecological disorders: a Mendelian randomization study
Source: Front Endocrinol (Lausanne). 2023 Nov 21;14:1161356. doi: 10.3389/fendo.2023.1161356 (PMC10710168; doi:10.3389/fendo.2023.1161356)

Supplementary Material

Genetically predicted serum testosterone and risk of gynecological disorders: A Mendelian randomization study

**Benzheng Zhao^1^, Zhenpeng Wang^1^, Dongzhen Liu^1^, Songling Zhang^1*^**

*** Correspondence:** Songling Zhang: slzhang@jlu.edu.cn

# Supplementary Tables

**Table S1** **Mendelian randomization results of weighted median and MR-Egger methods about total testosterone on gynecological diseases.**

| **Outcome** | **NSNP** | **Weighted median** | | **MR Egger** | | **P_heterogeneity_** | **P_pleiotropy_** |
| --- | --- | --- | --- | --- | --- | --- | --- |
|  |  | OR (95%LCI-95%UCI) | P | OR (95%LCI-95%UCI) | P |  |  |
| **Ovarian cancer** | 125 | 0.917(0.769-1.094) | 0.259 | 0.898(0.747-1.081) | 0.337 | 0.046 | 0.849 |
| Serous | 125 | 0.933(0.763-1.140) | 0.437 | 0.919(0.744-1.136) | 0.495 | 0.068 | 0.780 |
| Mucinous | 125 | 0.746(0.496-1.120) | 0.220 | 0.735(0.45-1.119) | 0.157 | 0.429 | 0.219 |
| Endometrioid | 125 | 0.815(0.568-1.171) | 0.211 | 0.769(0.51-1.158) | 0.269 | 0.012 | 0.369 |
| Clear cell | 125 | 0.949(0.661-1.474) | 0.782 | 1.084(0.614-1.914) | 0.817 | 0.041 | 0.267 |
| **Endometrial cancer** | 131 | 1.593(1.339-1.894) | **<0.001** | 1.856(1.452-2.373) | **<0.001** | <0.001 | 0.098 |
| Endometrioid | 131 | 1.784(1.443-2.206) | **<0.001** | 1.823(1.348-2.466) | **<0.001** | <0.001 | 0.181 |
| Non-endometrioid | 127 | 1.461(0.905-2.359) | 0.120 | 1.703(0.729-3.978) | 0.221 | 0.268 | 0.913 |
| **Cervical cancer** | 81 | 1.000(0.998-1.002) | 0.494 | 1.001(0.988-1.005) | 0.878 | 0.342 | 0.843 |
| **Ovarian cyst** | 100 | 1.023(0.852-1.230) | 0.495 | 1.091(0.85-1.401) | 0.806 | <0.001 | 0.065 |
| **Endometriosis** | 100 | 0.876(0.698-1.099) | 0.332 | 0.847(0.606-1.183) | 0.252 | <0.001 | 0.722 |
| **POF** | 100 | 0.532(0.177-1.599) | 0.550 | 0.705(0.225-2.211) | 0.261 | 0.883 | 0.563 |
| **PCOS** | 100 | 2.346(1.200-4.587) | **0.023** | 2.364(1.14-4.901) | **0.013** | 0.896 | 0.219 |
| **Cervical polyp** | 75 | 1.000(0.998-1.002) | 0.586 | 1.001(0.997-1.006) | 0.913 | 0.086 | 0.458 |
| **Uterine fibroids** | 122 | 0.998(0.994-1.002) | 0.142 | 0.997(0.994-1.001) | 0.237 | 0.543 | 0.619 |

Abbreviations: NSNP, number of SNPs; p_heterogeneity_, p value of Cochrane’s Q value in heterogeneity test; p_pleiotropy_, p value of MR-Egger intercept.

**Table S2 Mendelian randomization results of weighted median and MR-Egger methods about bioavailable testosterone on gynecological diseases.**

| **Outcome** | **NSNP** | **Weighted median** | | **MR Egger** | | **P_heterogeneity_** | **P_pleiotropy_** |
| --- | --- | --- | --- | --- | --- | --- | --- |
|  |  | OR (95%LCI-95%UCI) | P | OR (95%LCI-95%UCI) | P |  |  |
| **Ovarian cancer** | 115 | 0.888(0.776-1.013) | 0.119 | 0.995(0.813-1.216) | 0.958 | 0.045 | 0.142 |
| Serous | 115 | 0.944(0.791-1.127) | 0.524 | 1.040(0.824-1.313) | 0.740 | 0.041 | 0.103 |
| Mucinous | 115 | 0.915(0.581-1.441) | 0.700 | 0.817(0.452-1.476) | 0.504 | 0.063 | 0.501 |
| Endometrioid | 115 | 0.941(0.695-1.273) | 0.692 | 0.877(0.576-1.335) | 0.541 | 0.158 | 0.841 |
| Clear cell | 115 | 0.591(0.370-0.945) | 0.028 | 0.741(0.425-1.292) | 0.293 | 0.509 | 0.935 |
| **Endometrial cancer** | 120 | 1.478(1.212-1.802) | <0.001 | 1.951(1.486-2.563) | <0.001 | <0.001 | 0.034 |
| Endometrioid | 120 | 1.420(1.130-1.783) | 0.003 | 1.854(1.353-2.540) | <0.001 | <0.001 | 0.154 |
| Non-endometrioid | 117 | 1.768(0.981-3.184) | 0.058 | 1.950(0.890-4.273) | 0.098 | 0.135 | 0.454 |
| **Cervical cancer** | 73 | 0.999(0.998-1.001) | 0.453 | 1.001(0.998-1.004) | 0.435 | 0.087 | 0.494 |
| **Ovarian cyst** | 114 | 1.015(0.857-1.202) | 0.867 | 1.033(0.789-1.352) | 0.815 | <0.001 | 0.606 |
| **Endometriosis** | 133 | 0.830(0.686-1.004) | 0.055 | 0.866(0.654-1.148) | 0.320 | <0.001 | 0.818 |
| **POF** | 114 | 0.538(0.194-1.490) | 0.233 | 0.577(0.169-1.969) | 0.382 | 0.379 | 0.476 |
| **PCOS** | 114 | 1.836(1.041-3.238) | 0.036 | 1.016(0.442-2.336) | 0.970 | 0.086 | 0.202 |
| **Cervical polyp** | 78 | 1.001(0.999-1.003) | 0.529 | 1.000(0.997-1.003) | 0.869 | 0.081 | 0.815 |
| **Uterine fibroids** | 113 | 1.000(0.997-1.003) | 0.910 | 1.001(0.997-1.006) | 0.619 | 0.033 | 0.438 |

**Table S3 Mendelian randomization results of weighted median and MR-Egger methods about dehydroepiandrosterone sulfate on gynecological diseases**.

| **Outcome** | **NSNP** | **Weighted median** | | **MR Egger** | | **P_heterogeneity_** | **P_pleiotropy_** |
| --- | --- | --- | --- | --- | --- | --- | --- |
|  |  | OR (95%LCI-95%UCI) | P | OR (95%LCI-95%UCI) | P |  |  |
| **Ovarian cancer** | 7 | 0.908(0.766-1.077) | 0.267 | 0.852(0.687-1.057) | 0.205 | 0.411 | 0.182 |
| Serous | 7 | 0.976(0.794-1.201) | 0.822 | 0.896(0.656-1.224) | 0.521 | 0.208 | 0.539 |
| Mucinous | 7 | 0.554(0.331-0.926) | 0.024 | 0.462(0.251-0.850) | 0.056 | 0.796 | 0.341 |
| Endometrioid | 7 | 0.755(0.523-1.091) | 0.135 | 0.750(0.474-1.188) | 0.275 | 0.527 | 0.506 |
| Clear cell | 7 | 1.104(0.660-1.847) | 0.705 | 0.862(0.444-1.673) | 0.679 | 0.791 | 0.159 |
| **Endometrial cancer** | 6 | 1.814(1.451-2.267) | <0.001 | 1.941(1.349-2.793) | 0.023 | 0.184 | 0.585 |
| Endometrioid | 6 | 1.765(1.357-2.297) | <0.001 | 1.913(1.159-3.158) | 0.064 | 0.071 | 0.572 |
| Non-endometrioid | 6 | 0.743(0.416-1.327) | 0.316 | 0.521(0.181-1.498) | 0.293 | 0.053 | 0.267 |
| **Cervical cancer** | 2 | - | - | - | - | 0.345 | - |
| **Ovarian cyst** | 7 | 0.985(0.850-1.142) | 0.845 | 1.078(0.900-1.291) | 0.452 | 0.295 | 0.143 |
| **Endometriosis** | 7 | 0.847(0.719-0.997) | 0.045 | 0.846(0.676-1.059) | 0.205 | 0.434 | 0.933 |
| **POF** | 7 | 0.321(0.138-0.745) | 0.008 | 0.229(0.079-0.670) | 0.043 | 0.375 | 0.140 |
| **PCOS** | 7 | 2.316(1.324-4.051) | 0.003 | 3.22(1.632-6.353) | 0.020 | 0.234 | 0.110 |
| **Cervical polyp** | 2 | - | - | - | - | 0.072 | - |
| **Uterine fibroids** | 7 | 0.998(0.994-1.001) | 0.226 | 0.999(0.995-1.004) | 0.804 | 0.418 | 0.327 |

**Table S4 Mendelian randomization results of weighted median and MR-Egger methods about sex hormone-binding globulin on gynecological diseases.**

| **Outcome** | **NSNP** | **Weighted median** | | **MR Egger** | | **P_heterogeneity_** | **P_pleiotropy_** |
| --- | --- | --- | --- | --- | --- | --- | --- |
|  |  | OR (95%LCI-95%UCI) | P | OR (95%LCI-95%UCI) | P |  |  |
| **Ovarian cancer** | 167 | 1.022(0.906-1.053) | 0.726 | 0.999(0.871-1.145) | 0.99 | 0.067 | 0.698 |
| Serous | 167 | 0.993(0.864-1.142) | 0.924 | 0.962(0.827-1.120) | 0.619 | 0.224 | 0.284 |
| Mucinous | 167 | 1.013(0.706-1.455) | 0.942 | 1.160(0.773-1.142) | 0.474 | 0.075 | 0.691 |
| Endometrioid | 167 | 0.889(0.692-1.142) | 0.358 | 0.934(0.682-1.280) | 0.672 | 0.004 | 0.967 |
| Clear cell | 167 | 0.976(0.667-1.428) | 0.899 | 1.457(0.993-2.138) | 0.056 | 0.48 | 0.146 |
| **Endometrial cancer** | 186 | 0.799(0.697-0.915) | 0.001 | 0.811(0.683-0.964) | 0.019 | <0.001 | 0.852 |
| Endometrioid | 186 | 0.773(0.657-0.910) | 0.002 | 0.782(0.645-0.950) | 0.014 | 0.001 | 0.551 |
| Non-endometrioid | 183 | 0.739(0.493-1.109) | 0.144 | 0.857(0.548-1.340) | 0.499 | 0.183 | 0.776 |
| **Cervical cancer** | 115 | 1.000(0.998-1.001) | 0.625 | 0.999(0.997-1.001) | 0.472 | 0.116 | 0.547 |
| **Ovarian cyst** | 176 | 0.966(0.842-1.108) | 0.624 | 0.943(0.823-1.080) | 0.398 | 0.394 | 0.473 |
| **Endometriosis** | 176 | 1.000(0.862-1.160) | 1.000 | 1.022(0.863-1.212) | 0.800 | 0.034 | 0.626 |
| **POF** | 176 | 1.099(0.532-2.274) | 0.798 | 1.920(0.843-4.373) | 0.122 | 0.152 | 0.155 |
| **PCOS** | 176 | 0.834(0.512-1.359) | 0.466 | 1.378(0.820-2.315) | 0.228 | 0.116 | 0.003 |
| **Cervical polyp** | 127 | 1.000(0.998-1.001) | 0.662 | 0.999(0.997-1.001) | 0.339 | 0.790 | 0.191 |
| **Uterine fibroids** | 170 | 0.997(0.994-1.000) | 0.028 | 0.998(0.995-1.001) | 0.139 | 0.251 | 0.699 |

**Table S5 Multivariable Mendelian randomization results of Body Mass Index and four testosterone related exposure factors on gynecological diseases.**

| **Exposure** | **Outcome** | **NSNP** | **Beta** | **SE** | **P** |
| --- | --- | --- | --- | --- | --- |
| BMI | Ovarian cancer | 52 | 0.203 | 0.072 | 0.005 |
| TT | Ovarian cancer | 70 | -0.080 | 0.062 | 0.200 |
| BMI | Serous ovarian cancer | 52 | 0.171 | 0.082 | 0.036 |
| TT | Serous ovarian cancer | 70 | -0.036 | 0.070 | 0.604 |
| BMI | Endometrial cancer | 54 | 0.595 | 0.097 | <0.001 |
| TT | Endometrial cancer | 71 | 0.403 | 0.085 | <0.001 |
| BMI | Endometrial cancer (endometrioid histology) | 54 | 0.657 | 0.116 | <0.001 |
| TT | Endometrial cancer (endometrioid histology) | 71 | 0.395 | 0.102 | <0.001 |
| BMI | Endometrial cancer (Non-endometrioid histology) | 54 | 0.304 | 0.247 | 0.218 |
| TT | Endometrial cancer (Non-endometrioid histology) | 71 | 0.353 | 0.215 | 0.101 |
| BMI | Endometriosis | 54 | -0.165 | 0.142 | 0.245 |
| TT | Endometriosis | 67 | -0.209 | 0.127 | 0.101 |
| BMI | Polycystic ovarian syndrome | 54 | 0.891 | 0.331 | 0.007 |
| TT | Polycystic ovarian syndrome | 67 | 0.280 | 0.297 | 0.345 |
| BMI | Ovarian cancer | 55 | 0.177 | 0.072 | 0.015 |
| Bio-T | Ovarian cancer | 79 | -0.138 | 0.056 | 0.014 |
| BMI | Serous ovarian cancer | 55 | 0.161 | 0.082 | 0.052 |
| Bio-T | Serous ovarian cancer | 79 | -0.103 | 0.064 | 0.108 |
| BMI | Clear cell ovarian cancer | 55 | 0.139 | 0.201 | 0.489 |
| Bio-T | Clear cell ovarian cancer | 79 | -0.533 | 0.156 | 0.001 |
| BMI | Endometrial cancer | 57 | 0.510 | 0.096 | <0.001 |
| Bio-T | Endometrial cancer | 83 | 0.422 | 0.074 | <0.001 |
| BMI | Endometrial cancer (endometrioid histology) | 57 | 0.559 | 0.111 | <0.001 |
| Bio-T | Endometrial cancer (endometrioid histology) | 83 | 0.460 | 0.086 | <0.001 |
| BMI | Endometrial cancer (Non-endometrioid histology) | 57 | 0.234 | 0.233 | 0.315 |
| Bio-T | Endometrial cancer (Non-endometrioid histology) | 83 | 0.427 | 0.181 | 0.018 |
| BMI | Endometriosis | 57 | -0.192 | 0.136 | 0.157 |
| Bio-T | Endometriosis | 80 | -0.053 | 0.106 | 0.615 |
| BMI | Polycystic ovarian syndrome | 57 | 0.822 | 0.334 | 0.014 |
| Bio-T | Polycystic ovarian syndrome | 80 | 0.443 | 0.261 | 0.089 |
| BMI | Mucinous ovarian cancer | 62 | 0.356 | 0.225 | 0.114 |
| DHEAS | Mucinous ovarian cancer | 2 | 0.521 | 0.409 | 0.203 |
| BMI | Endometrial cancer | 64 | 0.594 | 0.086 | <0.001 |
| DHEAS | Endometrial cancer | 2 | 0.165 | 0.159 | 0.300 |
| BMI | Endometrial cancer (endometrioid histology) | 64 | 0.599 | 0.097 | <0.001 |
| DHEAS | Endometrial cancer (endometrioid histology) | 2 | 0.129 | 0.178 | 0.468 |
| BMI | Endometriosis | 63 | -0.102 | 0.118 | 0.387 |
| DHEAS | Endometriosis | 2 | -0.119 | 0.216 | 0.583 |
| BMI | Premature ovarian failure | 63 | -0.499 | 0.441 | 0.258 |
| DHEAS | Premature ovarian failure | 2 | 0.678 | 0.809 | 0.402 |
| BMI | Polycystic ovarian syndrome | 63 | 1.080 | 0.337 | 0.001 |
| DHEAS | Polycystic ovarian syndrome | 2 | -0.392 | 0.618 | 0.526 |
| BMI | Endometrial cancer | 53 | 0.485 | 0.094 | <0.001 |
| SHBG | Endometrial cancer | 105 | -0.215 | 0.066 | 0.001 |
| BMI | Endometrial cancer (endometrioid histology) | 53 | 0.530 | 0.103 | <0.001 |
| SHBG | Endometrial cancer (endometrioid histology) | 105 | -0.246 | 0.073 | 0.001 |
| BMI | Polycystic ovarian syndrome | 53 | 0.960 | 0.319 | 0.003 |
| SHBG | Polycystic ovarian syndrome | 103 | -0.755 | 0.232 | 0.001 |

# Supplementary Figures

**Figure S1 Scatter plots for the causal association between total testosterone and gynecological diseases.**


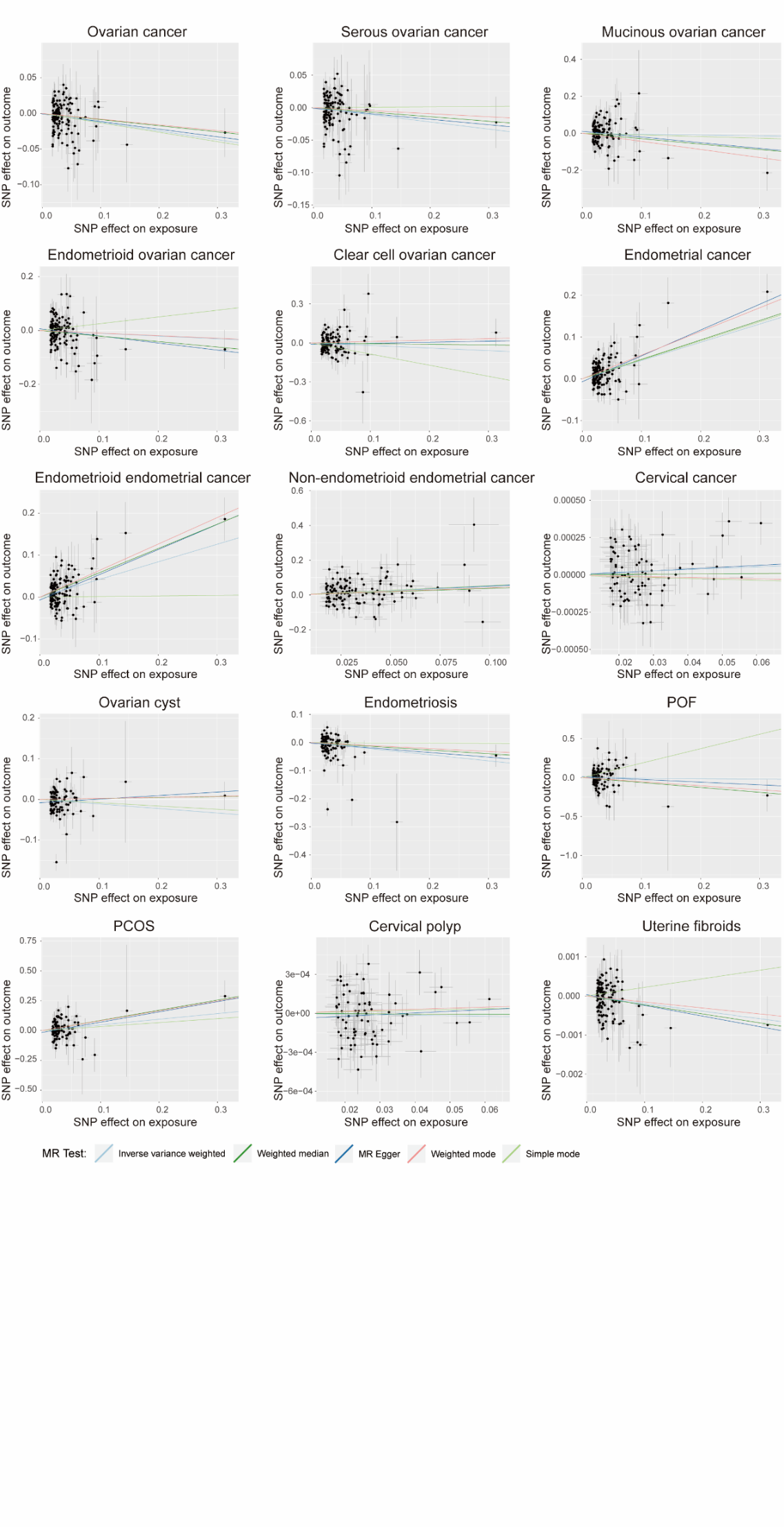


**Figure S2 Scatter plots for the causal association between bioavailable testosterone and gynecological diseases.**


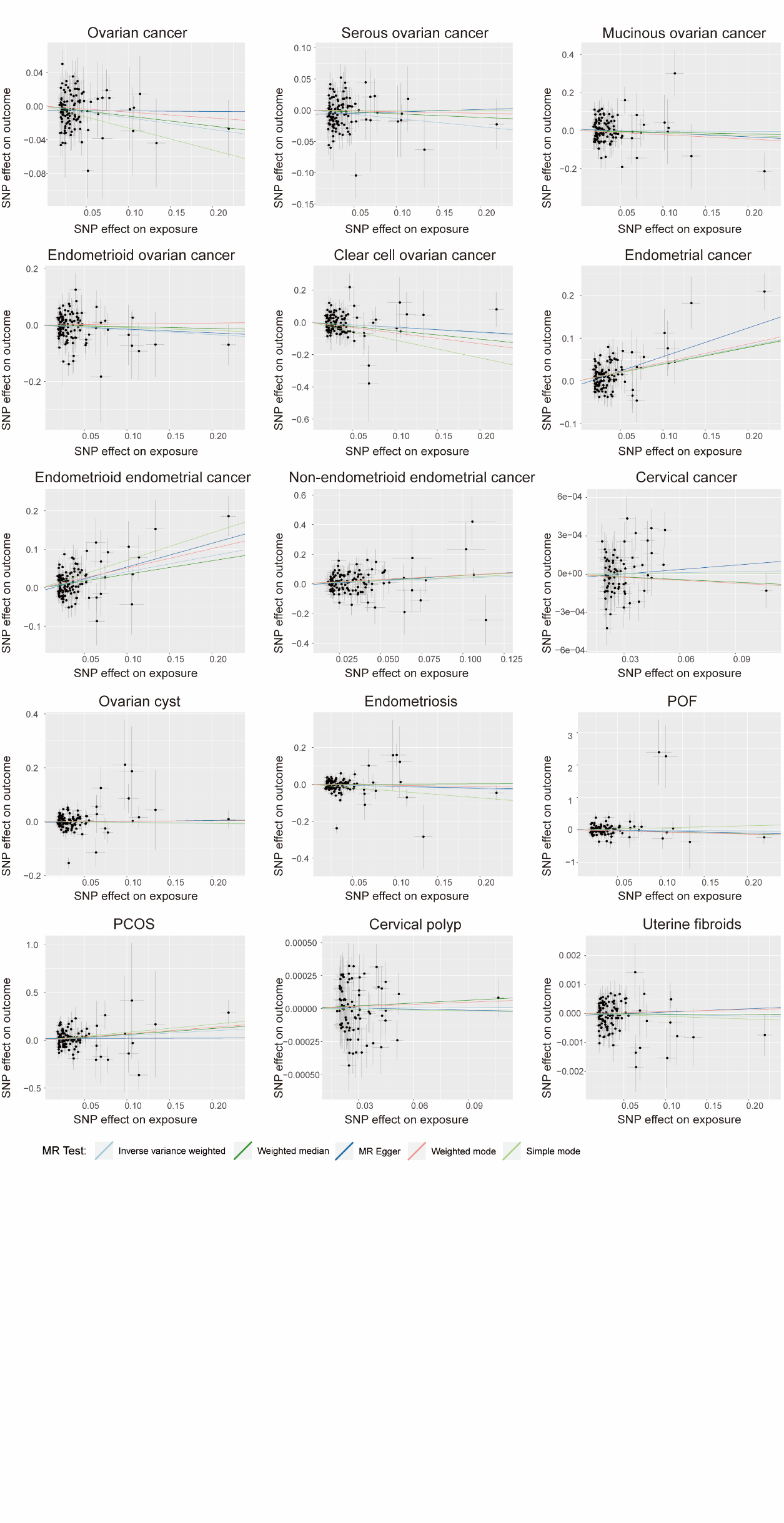


**Figure S3 Scatter plots for the causal association between dehydroepiandrosterone sulfate and gynecological diseases.**


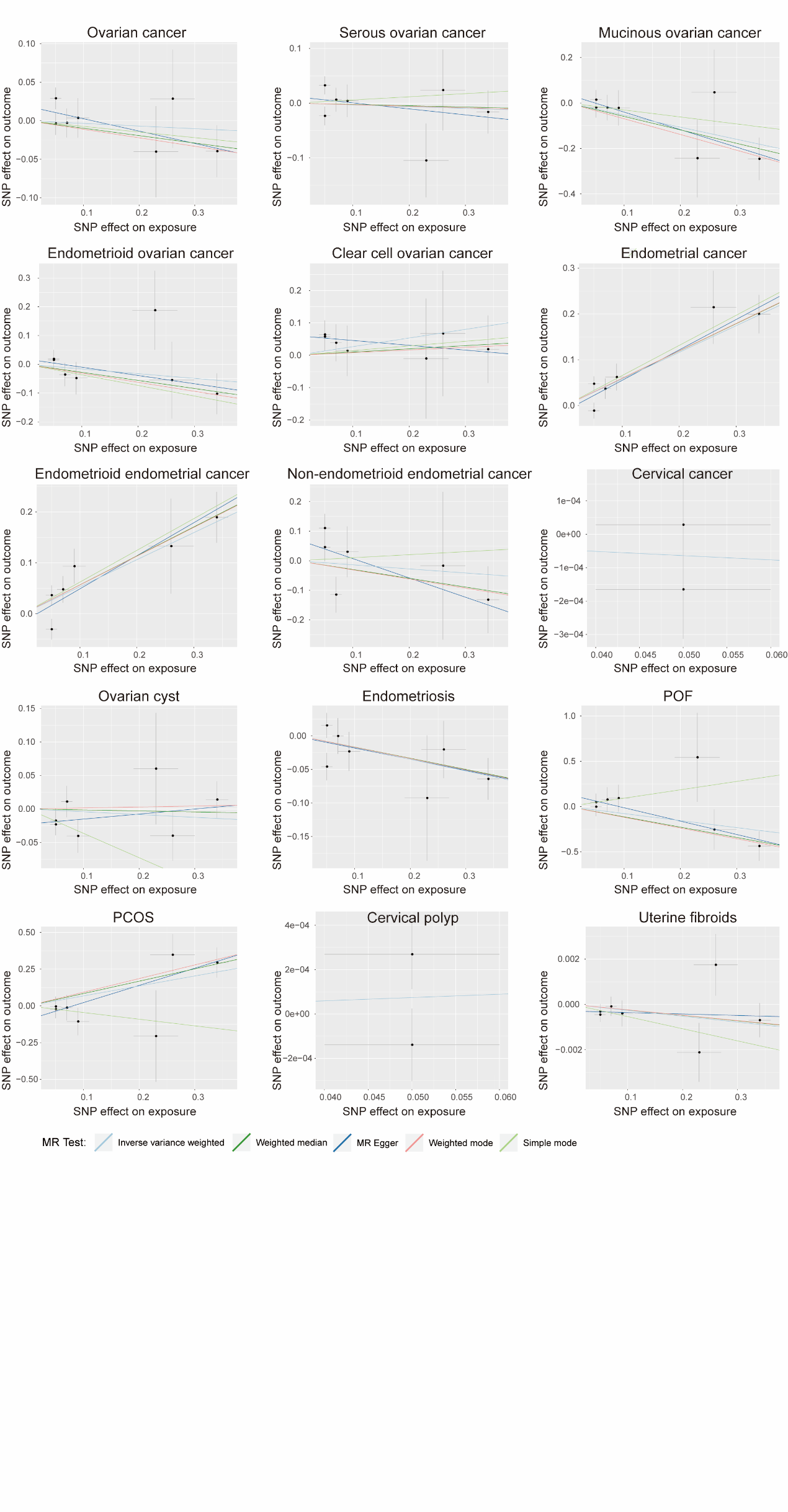


**Figure S4 Scatter plots for the causal association between sex hormone-binding globulin and gynecological diseases.**


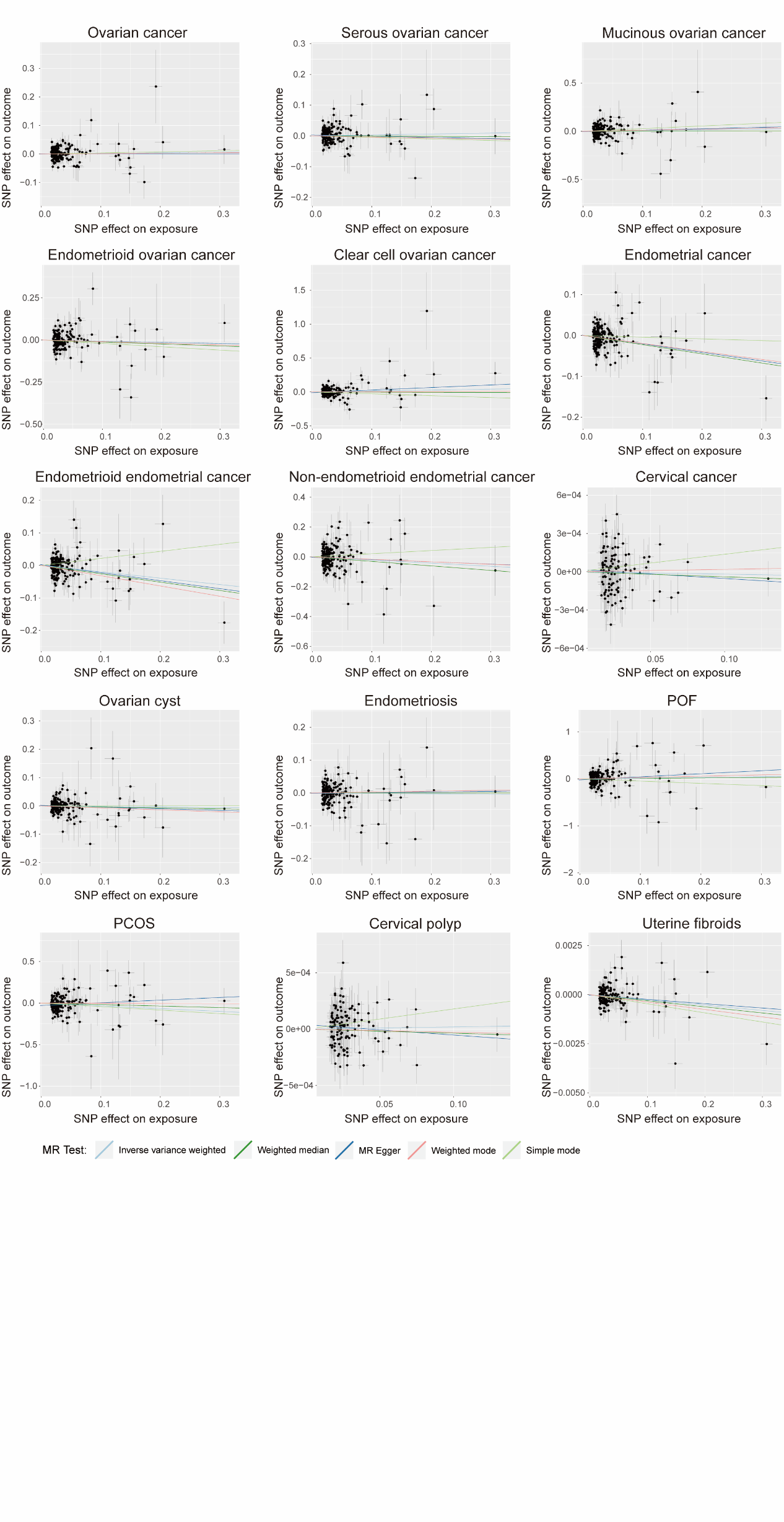

Supplement: Supplementary file 1 [file DataSheet_1.docx]
